# An automated segmentation approach to calibrating infantile nystagmus waveforms

**DOI:** 10.3758/s13428-018-1178-5

**Published:** 2019-03-11

**Authors:** Matt J. Dunn, Christopher M. Harris, Fergal A. Ennis, Tom H. Margrain, J. Margaret Woodhouse, Lee McIlreavy, Jonathan T. Erichsen

**Affiliations:** 1 School of Optometry and Vision Sciences, Cardiff University, Cardiff, UK; 2 Centre for Robotics and Neural Systems, Plymouth University, Plymouth, UK

**Keywords:** Eye movements, Calibration, Foveation

## Abstract

Infantile nystagmus (IN) describes a regular, repetitive movement of the eyes. A characteristic feature of each cycle of the IN eye movement waveform is a period in which the eyes are moving at minimal velocity. This so-called “foveation” period has long been considered the basis for the best vision in individuals with IN. In recent years, the technology for measuring eye movements has improved considerably, but there remains the challenge of calibrating the direction of gaze in tracking systems when the eyes are continuously moving. Identifying portions of the nystagmus waveform suitable for calibration typically involves time-consuming manual selection of the foveation periods from the eye trace. Without an accurate calibration, the exact parameters of the waveform cannot be determined. In this study, we present an automated method for segmenting IN waveforms with the purpose of determining the foveation positions to be used for calibration of an eye tracker. On average, the “point of regard” was found to be within 0.21° of that determined by hand-marking by an expert observer. This method enables rapid clinical quantification of waveforms and the possibility of gaze-contingent research paradigms being performed with this patient group.

Infantile nystagmus (IN) is a repetitive, primarily horizontal movement of the eyes. The condition usually develops within the first six months of life, causing ocular oscillations that are both constant and incurable. IN is characterized by its “waveform”—namely, the position-versus-time relationship with which the eyes move. An example of an IN waveform is given in Fig. 1, showing both the slow and quick phases.

**Fig. 1:**
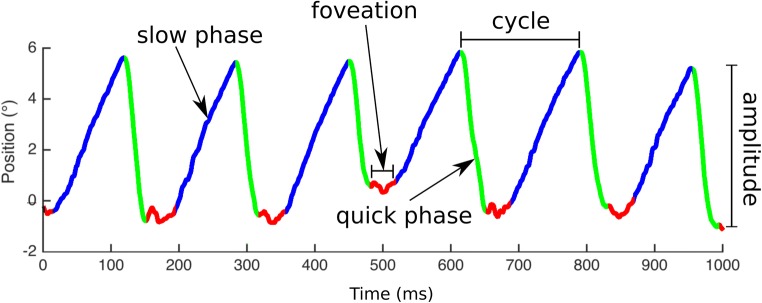
Example of a nystagmus waveform. An upward deflection of the trace indicates a rightward eye movement; a downward deflection is a leftward movement. Nystagmus intensity is calculated as the product of frequency and amplitude

The regular cycles of most adult IN waveforms contain periods during which the eye velocity (change in gaze angle over time) is significantly lower. These are known as “foveation” periods, and their duration, velocity, and position variability across individuals are correlated with the underlying visual acuity (VA) deficit (Abadi & Worfolk, 1989; Bedell, White, & Abplanalp, 1989; Cesarelli, Bifulco, Loffredo, & Bracale, 2000).

Retinal imaging demonstrates that foveation periods *usually* coincide with the times at which the fovea is directed toward the object of regard (Felius et al., 2011), suggesting that foveations play an important role in visual perception in IN. As the “point of regard,” foveations can provide reference points against which eye-tracking systems can be calibrated.

Calibrating an eye tracker typically involves serial presentation of gaze targets at known locations in space. The output from the eye tracker at each of these locations is regressed against the known target locations, which provides a reference for converting the output signal into an estimated gaze angle (Harris, Hainline, & Abramov, 1981). It is usually preferable to calibrate prior to starting a recording session, since this allows for live output of eye position coordinates (in degrees), as well as facilitating human–computer interaction and gaze-contingent stimulus presentation.

The standard method for calibrating eye-tracking systems requires the user to look directly at visual targets displayed sequentially at known locations. Typically, the system waits until the gaze is almost stable before recording the gaze position for each calibration point. In most individuals with nystagmus, the eyes are never stable enough to be automatically accepted as fixations. Although it is usually possible to manually override the system (i.e., to force acceptance of eye position, regardless of ongoing movement), doing so introduces a potential calibration inaccuracy, since the gaze could be at any point along the nystagmus waveform at the time of manual override. Such inaccuracies may be tolerable for some applications. However, if we wish to guarantee maximum accuracy in clinical eye movement data, it is necessary to ensure that only the visual axis is used for calibration.

At present, accurately calibrating an eye tracker in the presence of nystagmus is a time-consuming process, requiring an expert observer to manually select the foveation periods from an eye trace and calibrate post hoc (Dell’Osso & Abel, 2006)*.* Alternatively, the operator may opt to ignore foveations altogether and simply use the average eye position from the entire eye trace for the duration during which the calibration target was presented. Although this is likely to be biased *toward* the foveation periods (since the eyes typically spend a larger proportion of time at or near foveation), it is an inherently inaccurate measure of the intended angle of gaze. Nystagmus waveforms with amplitudes of up to 15.7° have been reported (Abadi & Bjerre, 2002). Therefore, using the whole waveform to calibrate has the potential to introduce significant inaccuracies.

The ideal solution to this problem would be an automated calibration method based on the foveation periods of the waveform. However, foveation periods are typically defined by eye speed in degrees per second (a value that cannot be known until *after* the data are calibrated). Therefore, a different approach must be taken. Theodorou, Clement, Taylor, and Moore (2015) described a method that utilizes the linear relationship between the duration of saccades in the eye trace and their amplitude, since the quick phases of nystagmus follow the main sequence of saccades (Abadi & Worfolk, 1989). Although this solution is able to provide nystagmus amplitude and gaze *velocity* values, it does not give any information about *absolute* eye position, which is required in order to perform a complete calibration.

By definition, *foveations* in a nystagmus waveform are regular reductions in velocity, during which the fovea is generally directed toward the point of regard. The actual parameters that define the start and end of a foveation period are somewhat ambiguous; the exact definition (and whether it should be defined at all) remains a subject of debate. Westheimer and McKee (1975) found that VA in normally sighted individuals is not degraded by retinal image motion less than 2.5°/s, whereas Barnes and Smith (1981) identified a significant reduction in VA when subjects viewed visual targets moving at 3°–4°/s. A study by Chung and Bedell (1996), in which nystagmoid image motion was simulated in normally sighted individuals, showed that VA was significantly degraded when the retinal image velocity exceeded 3°/s for simulated foveation periods of 40–100 ms, whereas when the duration of the simulated foveation was reduced to 20 ms, 5°/s was the critical velocity at which VA worsened (as compared to nystagmoid motion of a lower velocity). This velocity criterion might reasonably be used to define foveation periods (although nystagmus-induced retinal image motion does not degrade VA in adults with IN; Dunn et al., 2014). Abadi and Worfolk (1989) arbitrarily defined foveation as an ocular velocity of less than 10°/s in a study comparing VA to foveation duration. Many publications since 1992 have settled on a threshold of 4°/s to define foveation periods (e.g., Bifulco, Cesarelli, Loffredo, Sansone, & Bracale, 2003; Cesarelli et al., 2000; Dell’Osso, van der Steen, Steinman, & Collewijn, 1992; Jones et al., 2013; Wiggins, Woodhouse, Margrain, Harris, & Erichsen, 2007). In addition, the definition of “foveation” often includes a positional constraint, according to which successive foveations must lie (for example) within ± 0.5° of one another (Dell’Osso & Jacobs, 2002; Dell’Osso et al., 1992). Foveations are also typically expected to exceed 7 ms in duration (Dell’Osso & Jacobs, 2002; Felius et al., 2011).

One of the difficulties with the current definition of “foveation” (apart from being impossible to calculate automatically prior to calibration) is that a fixed criterion is typically applied to all of the participants in a single study, despite the idiosyncratic and wide range of waveform dynamics observed in individuals with IN. Figure 2a shows an example of a (calibrated) recording from an individual with high-intensity nystagmus (participant P006 in the present study; mean intensity = 34.9°/s). Using a fixed foveation velocity threshold of 15°/s for a minimum duration of 7 ms, only two foveations are to be found in the 3-s recording. At the (more commonly used) 4°/s threshold, no foveations are found at all. Figure 2b shows another individual (participant P010; intensity = 5.3°/s), with foveations detected using the same foveation velocity threshold as in Fig. 2a (15°/s).^1^

**Fig. 2:**
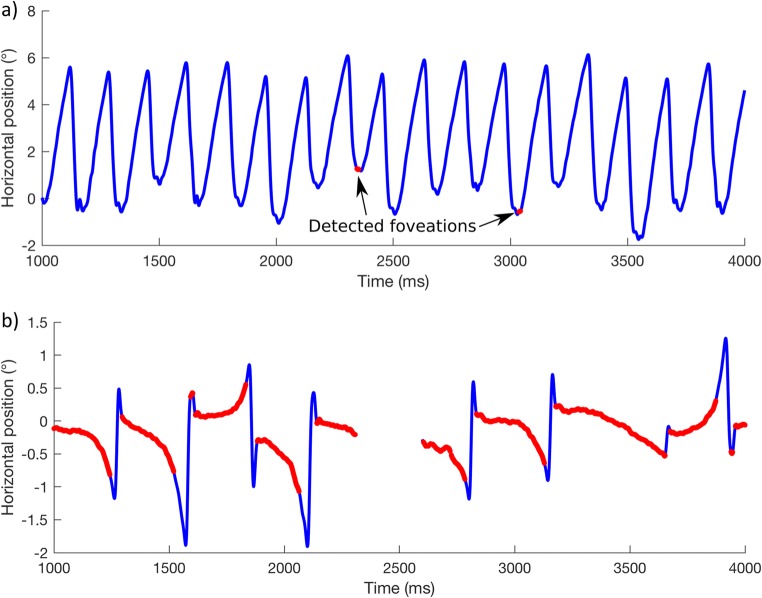
Nystagmus waveforms from two individuals with foveations detected (red) using the same *foveation velocity threshold* of 15°/s. **a** Participant P006 from the present study. **b** Participant P010

In Fig. 2b, we see that large portions of each cycle of the waveform are detected as foveations. Although one might reasonably conclude that the individual shown in Fig. 2b has “better foveation characteristics” than are shown in Fig. 2a, the parts of the waveform highlighted in each case cannot be said to represent the slowest portion of the waveform, nor are these portions both sufficient and accurate enough for reliable calibration of an eye tracker. Clearly, it is not appropriate to define foveation with a “one size fits all” criterion. Chung and Bedell (1996) suggested that the foveation velocity threshold ought to be different for each individual, set in relation to a fixed foveation duration. The approach set out in this article does not set a maximum velocity threshold and allows foveation duration to vary for each nystagmus cycle.

As well as relying on a predetermined velocity threshold, the current methods for detecting foveations typically use a position criterion—that is, all foveations must lie within a positional range, or they are rejected. This additional constraint may be useful when identifying foveation periods for the calibration of an eye tracker, but it does not give a true picture of foveation position variability. Our method does not impose such a constraint, allowing us to determine the actual positional variability of foveations in any given individual.

Felius et al. (2011) described a foveation detection method that uses a 4-s moving time window, in which the foveation position criterion is redefined at each time window based on the average eye position in the 4-s window. This goes some way toward enabling an objective, real-time view of where an individual with nystagmus is looking, but removing the position criterion altogether would be preferred, since this would allow the examiner to know where a patient was actually looking at *every* foveation. Manual calibration of nystagmus eye traces is both time-consuming and requires expertise. Automated foveation detection and nystagmus analysis would make clinical assessment more practicable.

The aim of the present study was to develop and validate a method for automating the detection of foveations in IN eye movement data, providing accurate coordinates that could be used to calibrate an eye tracker, thus enabling gaze-contingent research paradigms to be performed in this patient group. The method we employ separates the nystagmus signal into its component parts (quick phases and slow phases), which in turn provides the basis for automatic analysis of the properties of the entire nystagmus waveform, for example in a clinical setting.

## Method

The investigation was carried out in accordance with the Declaration of Helsinki; informed consent was obtained from all participants after explanation of the nature and possible consequences of the study. Ethical approval was granted by the Cardiff School of Optometry and Vision Sciences Research Ethics Audit Committee.

Eighteen individuals with early-onset nystagmus were recruited from the Cardiff Research Unit for Nystagmus cohort. The diagnosis of IN, as reported by the participant or by their ophthalmologist, was investigated by an optometrist using ophthalmoscopy, slit-lamp examination, optical coherence tomography, and a detailed family history. High-speed eye movement recordings from the present study were assessed to aid in the diagnosis of IN. Accelerating slow phases were an essential criterion for the diagnosis of IN (Abadi & Bjerre, 2002). Any participants whose data quality was not sufficient for the analysis (due to dropped samples) were also excluded post hoc. On these bases, six participants were excluded:

- Three individuals were excluded due to having fusion maldevelopment nystagmus syndrome (FMNS). Foveations are a hallmark feature of IN only, so calibration by foveation detection would not be possible in FMNS.
- One individual was excluded due to having acquired downbeat nystagmus (as above, acquired nystagmus waveforms do not contain foveation periods).
- One individual was excluded due to having nystagmus that was only present in rightward gaze (there was no nystagmus to analyze in other gaze positions).
- One individual was excluded due to having poor data capture (< 85%).

Twelve participants remained to take part in the study.

Participants were seated in a room, lit at ~ 1.78 log cd/m^2^, 2 m from a GDM-F520 21-in. CRT monitor (Sony Electronics Inc. San Diego, CA, USA). For the present study, eye tracking was performed monocularly at 1000 Hz using an EyeLink 1000 (SR Research, Ottawa, ON, Canada). The chin and head were supported by a rest. Participants wore their habitual refractive correction (if any), and the nondominant eye was patched. In the case of equidominance, the right eye was calibrated by default. Due to the high prevalence of strabismus in IN, it is essential to calibrate monocularly (i.e., with the nontest eye occluded). This prevents the patient from alternating fixation during the calibration procedure.

To have enough information to correctly scale an eye trace in two dimensions (i.e., horizontally and vertically), it is necessary to calibrate to multiple known locations in space. Any number of locations can be used, depending on the level of accuracy required. For the present study, a five-point calibration grid was used. Participants were instructed to fixate a simple black cross target subtending 0.3° × 0.3° on a midgray background. Targets were presented sequentially at ± 5° horizontally and ± 3° vertically (relative to the center of the screen). Such a narrow range of targets provides a greater challenge for calibration than do wider spacings, because any inaccuracies introduced by selecting the wrong portion of the waveform would have a greater impact on calibration accuracy. For this reason, we used a narrow range here in order to convince ourselves that our approach is robust. Each target was displayed for 10 s. For the calibration procedure, the first 300 ms at each fixation point were not analyzed, to give ample time to take up fixation of each target. For a five-point calibration, this procedure takes 50 s. The processing time for each calibration point depends on the hardware used; the process takes approximately 21 s for each 10-s segment using a MacBook Pro with an Intel Core i5 with a 2.6-GHz processor and 8 GB of RAM.

### Waveform analysis

To find the *slowest* period of each nystagmus cycle (i.e., the *foveations*), it is necessary to perform the following steps:

1. Filter and preprocess the eye-tracking data
2. Divide the nystagmus waveform into cycles
3. Distinguish quick phases from slow phases
4. Find the slowest part of each slow phase

Software to perform these tasks was written in MATLAB (The MathWorks, Natick, MA, USA) and is available to download for free (through a link provided at the end of this article).

First, any gaps in the eye-tracking signal of ≤ 25 ms are interpolated using cubic splines. Next, the eye movements associated with blinks are cleaned, by removing 75 ms on either side of any remaining gaps in the data. This removes spikes in eye position associated with tracking artifacts or blink-related movements.

The eye position signal is filtered using a generalized Savitzky–Golay filter (Dai, Selesnick, Rizzo, Rucker, & Hudson, 2017), and eye speed is also calculated by the method described by Dai et al. Note that, in uncalibrated data, all data are in arbitrary units. The primary axis of nystagmus (i.e., horizontal or vertical) is determined by finding the axis with the highest standard deviation of the position signal. The waveform is next split into cycles, using the method described by Pasquariello et al. (2010), which finds peaks in the waveform. Quick phases (saccades) are then detected using the algorithm designed by Pander, Czabański, Przybyła, and Pojda-Wilczek (2014). This saccade detection algorithm has the advantage of not requiring a precalibrated signal. Slow phases are considered to be any times when a saccade is *not* occurring.

### Foveation detection

The present algorithm looks for foveations in complete cycles only (i.e., those that do not contain any blinks). Since the purpose of the algorithm is to locate the “point of regard” for calibration of an eye tracker, it is not necessary to detect *every* foveation, but rather to be sure that those foveations that are detected are identified correctly. For each complete cycle, the total duration of the slow phases within that cycle is calculated (one cycle may contain multiple slow phases, but usually there is only one). The algorithm next looks for a foveation period lasting 10% of the total slow phase duration within that cycle, by determining the mean eye velocity (in arbitrary units) at each possible window of foveation (i.e., during a slow phase in the cycle). The foveation period for that cycle is the time window with the lowest mean velocity. Note that foveations are also permitted up to *one* foveation duration *after* the peak of a current cycle, to allow for foveations occurring at the cycle boundary (as is often the case in pendular waveforms). Foveations shorter than 7 ms are disregarded (Dell’Osso & Jacobs, 2002; Felius et al., 2011).

### Calibration procedure

For the data collected in the present study, from all of the foveation data obtained for each of the five calibration locations, the median gaze position during all detected foveation periods was calculated, resulting in a single coordinate pair for each calibration location, representing the “point of regard.” Using the median rather than the mean reduces the effect of any outliers on the selected coordinates, as well as biasing the chosen position toward the slower portion of each of the foveation periods. A two-dimensional polynomial regression was then calculated from these coordinate pairs and the known “true” coordinates of the calibration targets (in degrees). This calculation included a cross-talk term to account for rotation of the calibration field—that is, to account for any head tilt with respect to the stimulus monitor; see Harris et al. (1981). The regression coefficients were then saved to a calibration file. To calibrate data from the eye tracker, a transformation matrix was applied to each of the horizontal and vertical axes separately, using the coefficients stored in the calibration file.

Note that, since each calibration target location is analyzed separately, changes in waveform intensity and type at different gaze angles are accounted for. This is important, because the intensity and waveform of nystagmus can change with gaze angle (Abadi & Whittle, 1991).

### Verification

For each of the 12 participants, we compared the foveations detected by our method at one stimulus location (straight ahead) to the “gold standard”—having an expert with experience in interpreting nystagmus waveforms manually mark the beginning and end of all foveation periods in the eye trace, based on both the position and velocity channels from the eye trace. To eliminate bias, the manual marking was performed by a colleague with no prior knowledge of how the algorithm works (author F.A.E.). The positional precision and accuracy with which the “point of regard” was found was then compared between methods. As a control, we made the same comparisons to a “nonselective” automated approach in which we made no attempt to seek foveations, but simply took the median gaze position of the entire recording, including the quick phases.

The accuracy and precision of each method was assessed by comparing the distribution of gaze positions (in precalibrated units) across each of the eye-tracker samples identified, and by comparing the “points of regard” (i.e., the median gaze position across all identified samples) between methods.

## Results

Clinical findings for each participant are reported in Table 1.

**Table 1:** Clinical information about the participants in this study

Participant	Waveform in primary position	VA (logMAR)	Clinical diagnosis
P003	RPC	0.44	Idiopathic
P006	JL_EF_	0.36	Idiopathic
P009	JR_EF_	0.64	Idiopathic
P010	J_EF_ (PAN)	0.48	Idiopathic
P011	PP_FS_	0.60	Idiopathic
P013	JR_EF_	0.78	Idiopathic
P014	PJ (PAN)	0.42	Idiopathic
P015	BDJR	0.20	Idiopathic
P016	BDJR	0.52	Idiopathic
P017	BDJL	0.48	Idiopathic (fovea plana)
P018	PP_FS_	0.26	Unknown macular defect
P019	JR_EF_	0.16	Idiopathic

BDJ(R, L), bidirectional jerk (right, left); J(R)_EF_, jerk (right) with extended foveation; PAN, periodic alternating nystagmus; RPC, right pseudocycloid; PJ, pseudojerk; PP_FS_, pseudopendular with foveating saccades

**Fig. 3:**
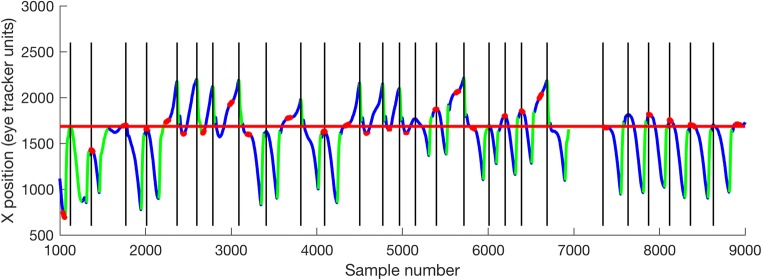
Example output from the waveform analysis procedure (for participant P014). Slow phases are shown in blue; quick phases are shown in green. Foveations are shown in red. The horizontal red line shows the median foveation position—that is, the position to be used for calibration. Vertical lines indicate cycle boundaries

Figure 3 shows an example of an uncalibrated eye trace with the waveform segmentation procedure applied. Regions highlighted in red are those identified as foveations. The “point of regard” is therefore determined as the median gaze position for all foveations (denoted in red).

**Fig. 4:**
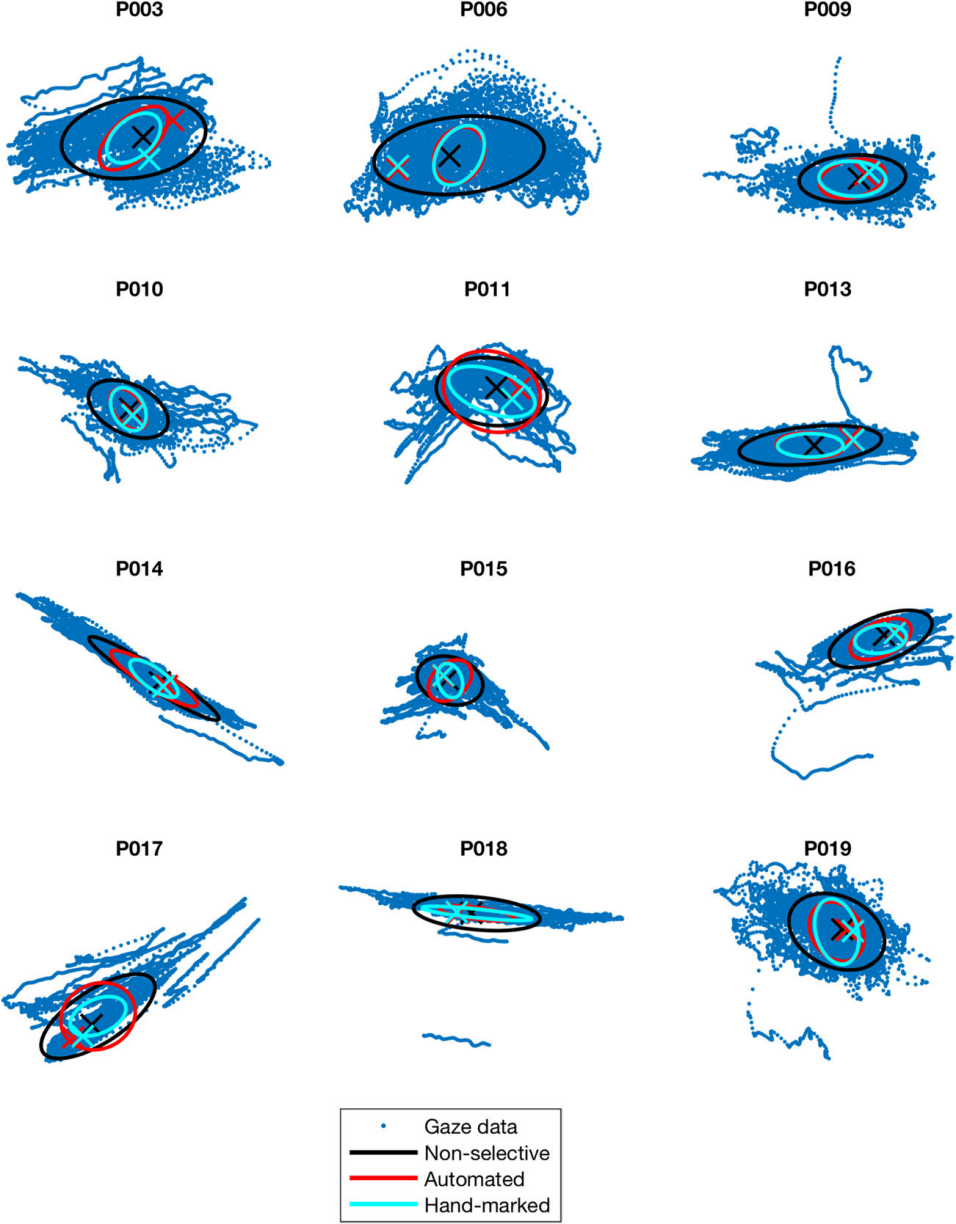
Plots showing the accuracy and precision of three methods of foveation detection. Blue dots indicate all gaze positions recorded over 10 s, in 2-D. Data are in uncalibrated eye-tracker units; hence, axes are not shown. Ellipses show the 68% confidence interval of all the mean gaze positions identified by each method. Crosses indicate the actual gaze position determined as the “point of regard.” Note that the “point of regard” is selected as the median of all identified samples, which may not necessarily lie within the 68% confidence intervals

Using color coding, Fig. 4 indicates, for each participant, the accuracy (crosses) and precision (ovals) of the algorithmic method, as compared to hand-marking by an expert observer and to the “nonselective” approach, in which all samples of the recording are considered candidates for calibration. In each case, the “point of regard” (cross)—that is, the gaze location to be used for calibration—is the median gaze position from each of the samples selected by that method.

Table 2 lists the total errors in calibration position found by the *algorithmic* and *nonselective* methods, as compared to hand-marking by an expert observer. To enable meaningful comparison, the values in Table 2 have been calibrated into degrees using the five-point calibration method described in the Method section.

**Table 2:** For each participant, total error in selected “point of regard,” as compared to those found using foveation periods hand-marked by an expert observer

Participant	Error compared to hand-marked foveation detection method		Difference
	Nonselective method	Algorithmic method	
P003	0.42° (12%)	0.79° (22%)	– 0.38° (11%)
P006	2.49° (40%)	0.04° (1%)	2.44° (39%)
P009	0.26° (11%)	0.09° (4%)	0.17° (7%)
P010	0.07° (3%)	0.02° (1%)	0.06° (3%)
P011	1.57° (17%)	0.58° (6%)	0.99° (11%)
P013	3.21° (24%)	0.25° (2%)	2.95° (22%)
P014	0.31° (3%)	0.35° (3%)	– 0.03° (0%)
P015	0.26° (6%)	0.04° (1%)	0.22° (5%)
P016	0.42° (11%)	0.08° (2%)	0.34° (9%)
P017	0.27° (10%)	0.24° (9%)	0.03° (1%)
P018	0.54° (11%)	0.04° (1%)	0.50° (10%)
P019	0.09° (8%)	0.05° (5%)	0.04° (4%)
**Mean**	**0.83° (13%)**	**0.21° (5%)**	**0.61° (10%)**

Positive values in the “Difference” column indicate that the *algorithmic* method was more accurate than the *nonselective* method in that participant. Errors are given both in absolute terms (calibrated visual degrees) and as a percentage of that participant’s median nystagmus amplitude during fixation

**Fig. 5:**
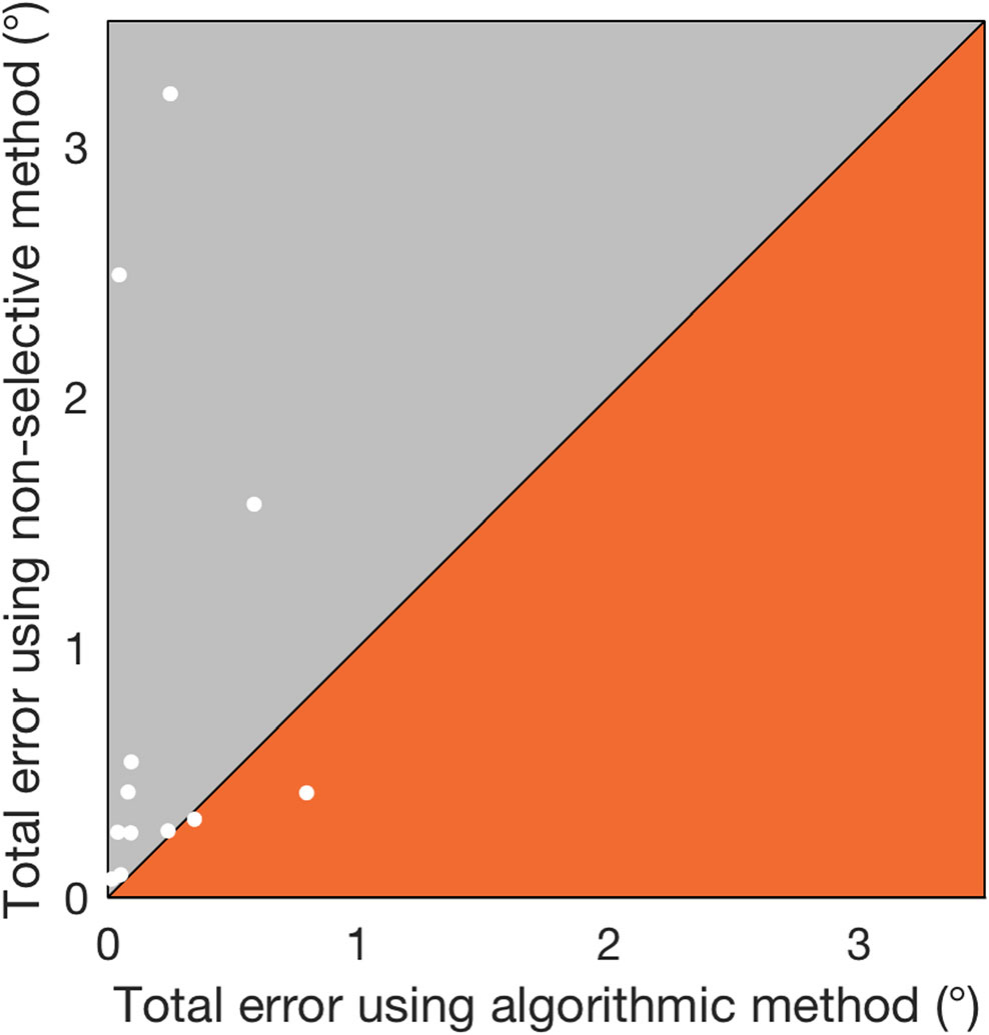
For each participant, total errors in “point of regard” selected by the *algorithmic* and *nonselective* methods, as compared to that found using foveation periods hand-marked by an expert observer. The data are the same as those shown in Table 2. The *y* = *x* line is shown, dividing the graph into two regions. Points above the line (in the gray region) indicate participants for whom our algorithm was more accurate than the nonselective method, and vice versa for points below the line (orange region)

Figure 5 shows the absolute error data from Table 2 graphically. Assuming that manual hand-marking of foveation periods represents the “gold standard” in foveation detection, the results in Table 2 indicate that our method more accurately located the “point of regard” in these participants (on average, to within 0.21°, or 5% of the nystagmus amplitude) than did the nonselective method.

**Fig. 6:**
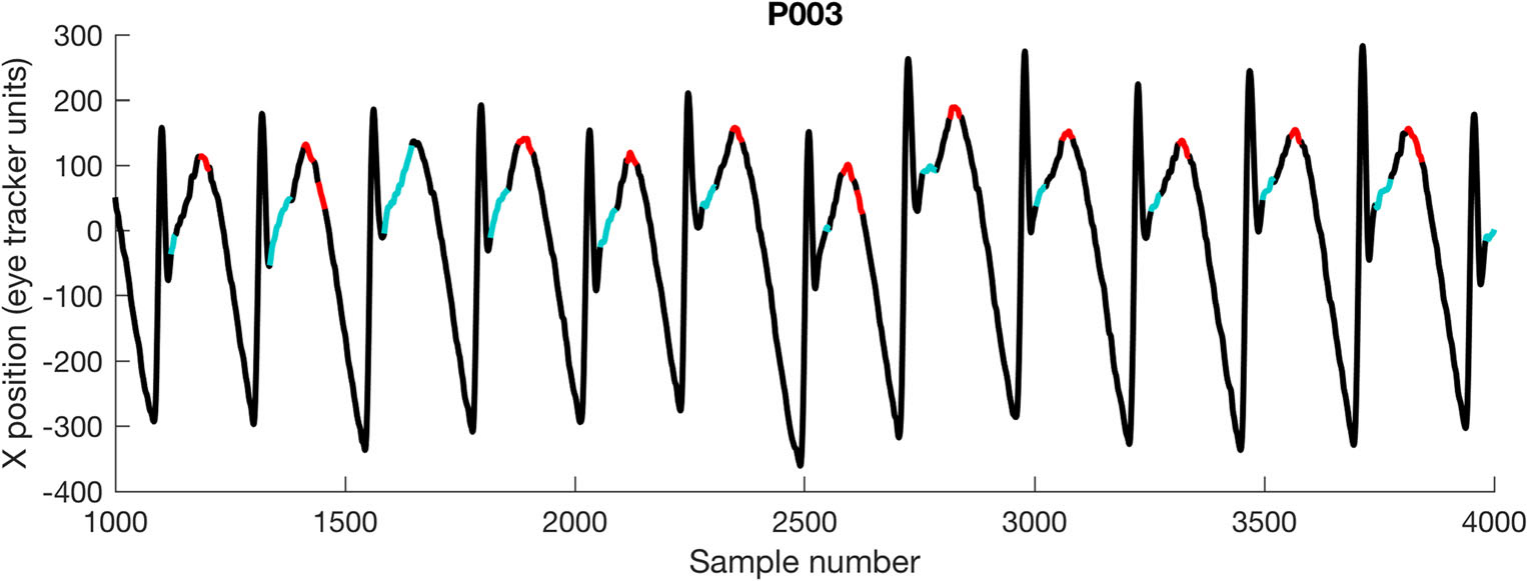
Comparison of the foveation periods detected in participant P003 by the algorithmic method (red), as compared to hand-marking by an expert observer (cyan)

It is worth noting the case of participant P003, for whom the nonselective method found the “point of regard” 0.38° more accurately than the algorithmic method. Figure 6 shows 3 s of the waveform from participant P003. This individual has an atypical *pseudocycloid* waveform with hypermetric quick phases. In this case, the algorithmic method has selected the region of the waveform typically considered to represent foveations in a pseudocycloid waveform (Dell’Osso & Daroff, 1975), and therefore—at least according to the original definition provided by Dell’Osso and Daroff—was in fact *more* reliable than hand-marking.

## Discussion

In the present study, we have described a novel automated segmentation method for detecting foveations in an uncalibrated IN waveform, providing the means to accurately calibrate an eye tracker in the presence of IN. The signal processing involved in reaching this goal conveniently parses the nystagmus waveform into its component parts, allowing for automated output of metrics relating to the entire waveform. Specifically, a fixed proportion of the slow phase of each cycle is identified as having the slowest velocity (in uncalibrated units), and the median position of these foveations is calibrated against the known position of the target. In this participant group, our algorithm produced “point of regard” measurements that were accurate, on average, to within 0.21° of the hand-marked foveations by an expert observer, and 0.61° more accurate than taking the median gaze position from the entire nystagmus waveform.

The method presented in this article is considerably closer to the current “gold standard” of foveation detection—that is, manual segmentation by an observer—than is using a nonselective method. The automation of this procedure enables eye-tracker calibration to take place *before* an eye movement recording session, rather than post hoc, as is typically the case. This in turn opens up the possibility of performing gaze-contingent eye-tracking studies in this patient group, as well as enabling human–computer interaction in consumer devices. With the advent of gaze-interactive computer systems, it is important that alternative calibration methods be available, to allow as many people as possible, including those with IN, to utilize these technologies.

It is worth clarifying that the main purpose of our algorithm is not to accurately and precisely identify the *times* of foveations, but to determine the “point of regard” for calibration of an eye-tracking system. Following calibration, it is possible to rerun our segmentation procedure using a classic foveation detection algorithm—that is, using a fixed *foveation velocity threshold*.

To perform a complete calibration, the segmentation procedure must be run on recordings containing attempted fixations on multiple target locations spanning the entire viewing area. The number of targets chosen, and their exact positions, will depend on the needs of the researcher/clinician, as will the exact method used to perform the calibration. For the purposes of validating our algorithm, we used Stampe’s (1993) calibration method. Of note, Rosengren, Nyström, Hammar, and Stridh (2019) have recently developed a complete calibration method for use in the presence of nystagmus, which uses our algorithm as a first step. We anticipate that, in future, our lab will calibrate nystagmus eye-tracking data using our algorithm in conjunction with Rosengren et al.’s method.

During a lengthy eye movement recording session, it is important to occasionally perform “drift corrections” to account for shifts in detected gaze position, which may occur as a result of head movements. A drift correction may be achieved by presenting a single fixation target and translating subsequent recorded gaze coordinates according to the offset from the original position, as determined by the algorithm.

**Fig. 7:**
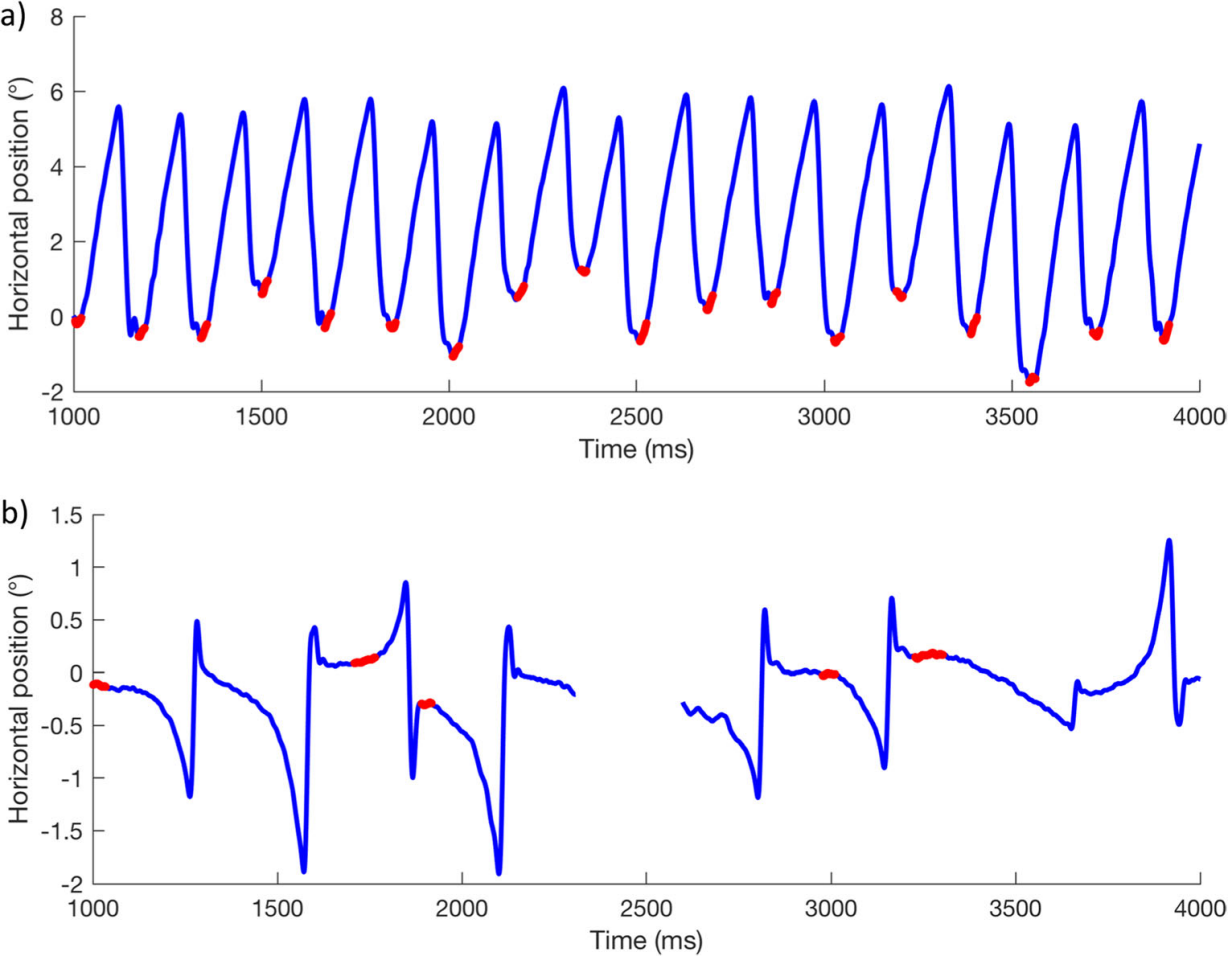
Examples of foveations detected (red) in the same nystagmus waveforms shown in Fig. 2, using the algorithmic method of foveation detection, in which the foveation velocity threshold can vary for each nystagmus cycle. As in Fig. 2, panel (**a**) shows participant P006, and panel (**b**) shows P010

Foveations are typically defined as the points in a nystagmus waveform at which the fovea is near the point of regard, which also happens to be when eye velocity is lowest. Our method does not require precalibrated data in order to find foveations in the waveform. In other words, the algorithm does not impose any constraints on position and is only concerned with *relative* velocity. Therefore, it should determine the “point of regard” for any individual with a waveform containing foveation periods. The classic method requires prior agreement on a fixed *foveation velocity threshold*, which may not be suitable for all patients (and, in any case, cannot be applied to uncalibrated data). To illustrate this point, Figs. 2 and 7 show the same data, but analyzed by two different methods—that is, using a fixed foveation velocity threshold and using our algorithmic method, respectively.

**Fig. 8:**
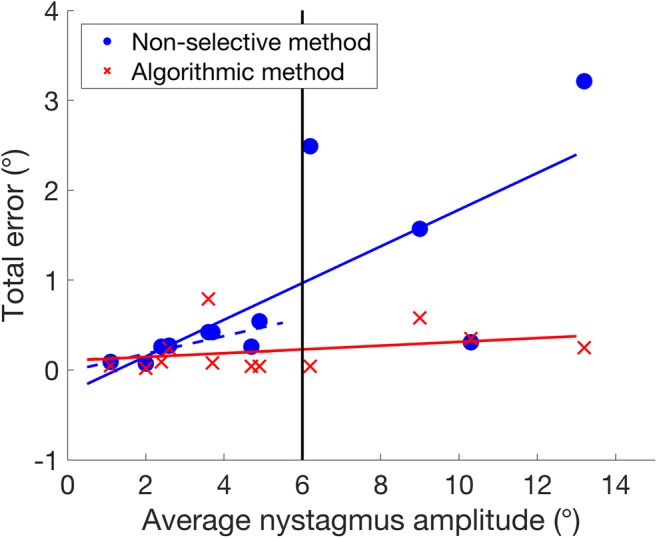
Impacts of nystagmus amplitude on the error of the *algorithmic* method (red crosses) and the *nonselective* method (blue circles). Solid lines show regressions of error against nystagmus amplitude for the entire data set. Dashed lines show regressions for participants with a nystagmus amplitude < 6° (vertical line). Note that the solid and dashed lines for the algorithmic method are superimposed

Note that, for both participants in Fig. 7, without specifying absolute thresholds, foveation data are detected in the majority of complete nystagmus cycles in both participants using a single automated method, despite their very different nystagmus intensities. This is desirable, since it provides enough data to determine the “point of regard” for calibrating an eye tracker, without inadvertently selecting large portions of the waveform. Thus, similar levels of accuracy can be obtained across a wide range of nystagmus intensities. In fact, a correlation analysis of the impact of nystagmus amplitude shows that the discrepancy between the algorithmic method and the hand-marked foveations is not significantly affected by amplitude (*p* = .319), whereas the error of the nonselective method is highly correlated with amplitude up to about 6° (*p* = .017), above which the size of the error becomes highly variable (see Fig. 8). This is not surprising, given that the nonselective method includes the entire waveform.

The intended purpose of our foveation detection method is to provide the means to rapidly and accurately calibrate eye-tracking data. Note, however, that foveation properties could also be assessed on the basis of the mean foveation velocity threshold obtained by our method (as opposed to *foveation duration*, as would be found using a fixed foveation velocity threshold).

The case of participant P003 (see Fig. 6) highlights the potential for ambiguity and disagreement in defining foveation periods. In this case, our algorithm found the portion of the waveform with the slowest velocity, which is also that portion originally defined by Dell’Osso and Daroff (1975), as being the foveation position in a *pseudocycloid* waveform. Nevertheless, this participant’s waveform was atypical, due to the presence of apparently hypermetric quick phases. Using retinal imaging (such as that used by Felius et al., 2011), it might be possible to determine the true foveation position in such cases.

**Fig. 9:**
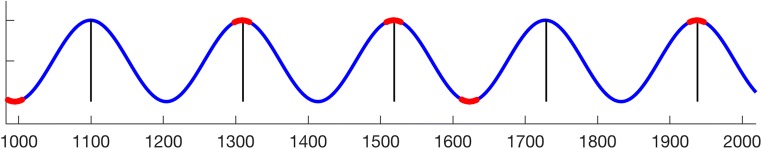
Simulation of a *pure pendular nystagmus waveform*, showing the detected cycle boundaries (black) and foveation positions (red)

The case of *pendular* IN also deserves some discussion. From eye movement data alone, it is impossible to know whether the peaks or troughs of these waveforms represent the “point of regard.” Arguably, foveations may exist on both “sides” of a pendular waveform. Although none of the participants in the present study had pendular nystagmus, we have confirmed the behavior of the algorithm with simulated data (modeled as a sine wave; see Fig. 9), as well as by examining data from other labs. In both cases, the algorithm finds foveations on both sides of the waveform. Since the “point of regard” is based on the *median* of all detected foveation positions, the end result is that the “point of regard” is found at whichever side of the waveform was selected most often.

Here we have described an algorithm to segment IN waveforms and to find, on the basis of foveations, the “point of regard,” which could be used to calibrate an eye tracker. Other forms of nystagmus, such as FMNS and acquired nystagmus, do not contain foveation periods. In these cases, it may be possible to find the “point of regard” by using the gaze position immediately following quick phases.

Any eye movement analysis algorithm relies on first obtaining reasonable eye movement recordings. IN is associated with a wide range of visual system pathologies, such as aniridia, albinism, microphthalmia, and colobomas (Holmström, Bondeson, Eriksson, Akerblom, & Larsson, 2013), any of which can present a challenge to the collection of accurate data with pupil-based eye trackers. It is worth considering in these situations whether an alternative eye-tracking system, such as a limbal tracker or scleral search coil, might be more appropriate. As long as gaze position data can be obtained, the present algorithm should be applicable.

The method presented here is a completely automated method for detecting IN foveations for calibration of an eye tracker. It is rapid, objective, accurate, and precise, without assuming similar nystagmus characteristics between individuals. Although in the present study we used an EyeLink 1000 eye tracker with a 1000-Hz capture rate in both the horizontal and vertical axes, this method can be applied to data from other eye trackers, although a reasonable sampling rate (e.g., ≥ 200 Hz; Leigh & Zee, 2006) is required in order to encompass the dynamics of the quick phases.

## Footnotes

1 Note that eye speed may be defined either as the velocity in a single axis (i.e., the horizontal or vertical eye-tracker channels) or as the total change in 2-D gaze position from sample to sample. We define eye speed in two dimensions, to account for nystagmus waveforms that do not oscillate about a principal axis. This results in eye speed values that are slightly lower than those calculated uniaxially.
